# Comparison of the effects of adjuvant concurrent chemoradiotherapy and chemotherapy for resected biliary tract cancer

**DOI:** 10.1186/s12876-020-1171-1

**Published:** 2020-01-28

**Authors:** Hyera Kim, Mi Hwa Heo, Jin Young Kim

**Affiliations:** Division of Hematology and Oncology, Department of Internal Medicine, Keimyung University, Dongsan Medical Center, 56 Dalseong-ro, Jung-gu, Daegu, 41931 South Korea

**Keywords:** Biliary tract neoplasms, Adjuvant chemoradiotherapy, Adjuvant chemotherapy

## Abstract

**Background:**

Biliary tract cancers (BTC) have a poor prognosis even after curative resection because of frequent local and distant recurrences. Therefore, the importance of adjuvant therapy in BTC has been advocated to improve outcomes. However, the choice of adjuvant therapy is still controversial. The aim of this study was to compare the effects of adjuvant concurrent chemoradiotherapy (CCRT) and chemotherapy on resected BTC.

**Methods:**

We analyzed 92 patients who had curatively resected BTC and had received adjuvant CCRT or chemotherapy from January 2000 to December 2017 at Keimyung University Dongsan Medical Center.

**Results:**

Of the patients, 46 received adjuvant CCRT and 46 received adjuvant chemotherapy. The median recurrence-free survival (RFS) for the adjuvant CCRT and chemotherapy groups were 13.8 and 11.2 months (*p* = 0.014), respectively. The median overall survival (OS) for the adjuvant CCRT and chemotherapy groups were 30.1 and 26.0 months (*p* = 0.222), respectively. Adjuvant CCRT had significantly better RFS and numerically higher OS than did chemotherapy. For subgroups with no lymph node (LN) involvement (RFS *p* = 0.006, OS *p* = 0.420) or negative resection margins (RFS *p* = 0.042, OS *p* = 0.098), adjuvant CCRT led to significantly longer RFS and numerically higher OS than did chemotherapy. For multivariate analysis, the pattern of adjuvant treatment (chemotherapy vs. CCRT, *p* = 0.004, HR 2.351), histologic grade (poor vs. well, *p* = 0.023, HR 4.793), and LN involvement (*p* = 0.028, HR 1.912) were the significant prognostic factors for RFS.

**Conclusions:**

Our study demonstrated the superiority of adjuvant CCRT over chemotherapy for improving RFS in curatively resected BTC.

## Background

Biliary tract cancers (BTC) are a heterogeneous group of neoplasms that includes cholangiocarcinoma and gallbladder cancer [1]. These cancers have a poor prognosis of low five-year survival rates in the range of 5 to 15% [2]. R0 resection has been the most important factor for the successful treatment of patients with BTC [3, 4]. However, less than 30% are resectable diseases at presentation because BTC are close to the complex anatomy of the porta hepatis [5–7]. Even after satisfactory curative resection, resectable diseases have five-year survival rates between 20 and 50% [8] due to frequent local and distant recurrences [5, 6]. Furthermore, the frequency of positive resection margins has been reported to be anywhere from 9 to 74% after curative-intent surgery [9]. Therefore, the importance of adjuvant therapy in BTC has been advocated to improve survival outcomes [10, 11].

Adjuvant treatments, including chemotherapy, radiotherapy, and chemoradiotherapy, may decrease the recurrence rate and improve overall survival (OS). Several single-center retrospective studies have demonstrated a survival benefit of adjuvant therapy in resected BTC [10–12]. Horgan et al. reported a systemic review and meta-analysis of published studies that showed the greatest OS improvement being achieved, especially in patients with node-positive and margin-positive diseases, when adjuvant chemotherapy or chemoradiotherapy was administered [13].

Despite the better outcomes of adjuvant chemotherapy and chemoradiotherapy following radical resection, the choice of adjuvant therapy, whether it be chemotherapy or chemoradiotherapy, is still controversial [7]. The current National Comprehensive Cancer Network (NCCN) guidelines recommend all options, including observation, chemotherapy, and chemoradiotherapy, for resected BTC and state that more data are necessary to make firm conclusions [14]. Therefore, the aim of this study was to compare the effects of adjuvant CCRT and chemotherapy in order to investigate the possible recurrence-free survival (RFS) or OS benefit after radical resection for BTC.

## Methods

### Patients and treatments

Of the patients who had undergone radical resection for BTC from January 2000 to December 2017 at Keimyung University Dongsan Medical Center, we collected 92 patients treated with adjuvant CCRT or chemotherapy. The patients met the following inclusion criterion of having histologically confirmed, non-metastatic BTC, which was defined as tumors of the gallbladder and the intrahepatic, perihilar, and distal extrahepatic bile ducts but excluding the ampulla of Vater. We reviewed the medical records retrospectively for the following characteristics: age, gender, date of death or the last follow-up visit, date of recurrence, the Karnofsky performance status (KPS), the Charlson comorbidity index (CCI), tumor location, histologic features (e.g. histology, grade, lymphovascular invasion, and perineural invasion), pathologic stage based on criteria from the American Joint Committee on Cancer (AJCC), 7th edition, LN status, resection margin status, the preoperative carbohydrate antigen 19–9 (CA19–9) levels as tumor markers, pattern of recurrence, and adjuvant treatment including chemotherapeutic agents. The cut-off value of serum CA 19–9 level was defined as 37 U/ml. RFS was defined as the time from the date of pathologic diagnosis to the date of recurrence, or death. OS was measured from the date of pathologic diagnosis to the date of death.

Curative-intent surgery was performed on all patients in this study. The adjuvant CCRT or chemotherapy plans and schedules depended on the clinicians’ decisions. Of the 92 patients, 46 received adjuvant concurrent chemotherapy and external beam radiotherapy. Of these 46 patients, 21 received CCRT followed by chemotherapy. The concurrent chemotherapy regimens included oral 5-fluorouracil (5-FU), such as uracil-tegafur (1 patient, 2.2%), intravenous 5-FU (7 patients, 15.2%), 5-FU/leucovorin (21 patients, 45.7%), 5-FU/cisplatin (12 patients, 26.1%), gemcitabine (4 patients, 8.7%), and other (1 patient, 2.2%). The patients received 4000–5400 cGy of external beam radiation in 28–30 fractions over 5–6 weeks. The radiation fields were tumor beds and regional LN. Of the patients, 2 received delayed adjuvant therapy after 4 months but the others had started treatment within at least 3 months. CCRT was begun at a mean of 7.3 weeks after surgery. All patients completed the whole course of CCRT. Of the 92 patients, 46 received only adjuvant chemotherapy. The chemotherapy regimens included oral 5-FU, such as uracil-tegafur (21 patients, 45.7%), intravenous 5-FU/leucovorin (18 patients, 39.1%), 5-FU/cisplatin (4 patients, 8.7%), gemcitabine/cisplatin (2 patients, 4.3%), and gemcitabine (1 patient, 2.2%). Recurrence was classified into three patterns: locoregional recurrence, distant recurrence, and both. Locoregional recurrence was defined as recurrence in the tumor bed, anastomosis sites, or regional LN area. Distant recurrence was defined as recurrence in the non-regional LN area or in other organs. Most recurrences were clinically diagnosed by imaging studies, such as computed tomography or positron emission tomography, without pathologic confirmation. Patient follow-up was completed by March 2018.

This study was approved by the Institutional Review Board of the Keimyung University Dongsan Medical Center (DSMC 2018–08–045-001), which waived the requirement for written informed consent because of the retrospective nature of the study.

### Statistical analysis

The RFS and OS were calculated using the Kaplan-Meier method and were compared using the log-rank test. The Cox proportional hazard model was used for multivariate analyses to adjust for potential confounding factors. The results are presented as hazard ratios (HR) and 95% confidence intervals (CI). The chi-square test and Fisher’s exact test were used to compare the baseline characteristics among patients grouped by categorical variables. Continuous variables were compared using Student’s t-test. The level of critical significance was assigned at *p*-value < 0.05. Statistical data were analyzed with the IBM SPSS Statistics for Windows, version 20.0 (IBM Corp., Armonk, N.Y., USA).

## Results

### Patient characteristics

Table 1 describes the characteristics of the patients. All 92 patients met the inclusion criteria. The median age was 64.5 years (range, 34–81 years). There were 58 men (63%). KPS was ≥70 in 83 (90.2%) patients. The CCI, except for the malignancy score, was ≥3 in 42 (45.7%) patients. The tumor locations were the intrahepatic bile duct in 13 (14.1%), perihilar bile duct in 17 (18.5%), distal bile duct in 42 (45.7%), and gallbladder in 20 (21.7%) patients. The histology was adenocarcinoma in 86 (93.5%) patients. Four (4.3%) patients had well-differentiated tumors, 21 (22.8%) had poorly differentiated tumors, and the remaining (63%) patients had moderately differentiated tumors. The LN involvement was found in 54 (58.7%) patients. Forty-six (50.0%) patients had positive resection margins. According to the 2010 AJCC staging system, 14 (15.2%) patients were at Stage I, 37 (40.2%) were at Stage II, 28 (30.4%) were at Stage III, and 13 (14.1%) were at Stage IV. All Stage IV patients had M0 status, 10 patients had TxN1M0 (IVA) intrahepatic bile duct cancer, and 3 patients had TxN2M0 (IVB) GB cancer. Ten (10.9%) patients were pT1, 39 (42.4%) were pT2, 41 (44.6%) were pT3, and 2 (2.2%) were pT4. Sixty-four (69.6%) patients had lymphovascular invasion and 65 (70.7%) had perineural invasion. The preoperative CA19–9 levels were ≥ 37 U/ml in 50 (54.3%) patients.

**Table 1:** Characteristics of biliary tract cancer patients

Characteristic	Total (*n* = 92)	Adjuvant CCRT (*n* = 46)	Adjuvant chemotherapy (*n* = 46)	*P* value
Median age, years (range)	64.5 (34–81)	65.5 (44–81)	61.0 (34–80)	0.134
Men (%)	58 (63.0)	26 (56.5)	32 (69.6)	0.195
Performance (%)				
KPS ≥70	83 (90.2)	41 (89.1)	42 (91.3)	1.000
KPS < 70	9 (9.8)	5 (10.9)	4 (8.7)	
Comorbidities (%)				
CCI < 3	50 (54.3)	24 (52.2)	26 (56.5)	0.675
CCI ≥3	42 (45.7)	22 (47.8)	20 (43.5)	
Tumor location (%)				
Intrahepatic bile duct	13 (14.1)	6 (13.0)	7 (15.2)	0.756
Perihilar bile duct	17 (18.5)	10 (21.7)	7 (15.2)	
Distal bile duct	42 (45.7)	19 (41.3)	23 (50.0)	
Gallbladder	20 (21.7)	11 (23.9)	9 (19.6)	
Histology (%)				
Adenocarcinoma	86 (93.5)	43 (93.5)	43 (93.5)	1.000
Others	6 (6.5)	3 (6.5)	3 (6.5)	
Grade (%)				
Well	4 (4.3)	1 (2.2)	3 (6.5)	0.590
Moderate	63 (68.5)	32 (69.6)	31 (67.4)	
Poor	21 (22.8)	12 (26.1)	9 (19.6)	
NA	4 (4.3)	1 (2.2)	3 (6.5)	
LV invasion (%)				
Yes	64 (69.6)	29 (63.0)	35 (76.1)	0.213
No	25 (27.2)	15 (32.6)	10 (21.7)	
NA	3 (3.3)	2 (4.3)	1 (2.2)	
Perineural invasion (%)				
Yes	65 (70.7)	34 (73.9)	31 (67.4)	0.414
No	14 (15.2)	9 (19.6)	5 (10.9)	
NA	13 (14.1)	3 (6.5)	10 (21.7)	
pT stage (%)				
1	10 (10.9)	3 (6.5)	7 (15.2)	0.279
2	39 (42.4)	21 (45.7)	18 (39.1)	
3	41 (44.6)	22 (47.8)	19 (41.3)	
4	2 (2.2)	0 (0.0)	2 (4.3)	
LN involvement (%)				
Yes	54 (58.7)	26 (56.5)	28 (60.9)	0.672
No	38 (41.3)	20 (43.5)	18 (39.1)	
Pathologic stage (%)				
I	14 (15.2)	5 (10.9)	9 (19.6)	0.611
II	37 (40.2)	19 (41.3)	18 (39.1)	
III	28 (30.4)	16 (34.8)	12 (26.1)	
IV^a^	13 (14.1)	6 (13.0)	7 (15.2)	
Resection margin (%)				
Negative	45 (48.9)	16 (34.8)	29 (63.0)	0.007
Positive	47 (51.1)	30 (65.2)	17 (37.0)	
CA19–9 (%)				
< 37 U/ml	35 (38.0)	16 (34.8)	19 (41.3)	0.697
≥37 U/ml	50 (54.3)	25 (54.3)	25 (54.3)	
NA	7 (7.6)	5 (10.9)	2 (4.3)	
Recurrence (%)				
No	19 (20.7)	18 (39.1)	1 (2.2)	0.123
Locoregional	22 (23.9)	6 (13.0)	16 (34.8)	
Distant	36 (39.1)	13 (28.3)	23 (50.0)	
Both	15 (16.3)	9 (19.6)	6 (13.0)	

*CCRT* Concurrent chemoradiotherapy, *KPS* Karnofsky performance score, *CCI* Charlson comorbidity index, *LV* Lymphovascular, *LN* Lymph node

^a^All patients had M0 status, 10 patients had TxN1M0(IVA) intrahepatic bile duct cancers, and 3 patients had TxN2M0(IVB) GB cancers

Out of 92 patients, 46 received adjuvant CCRT and 46 received adjuvant chemotherapy. Median follow-up periods for both groups were 17.0 and 25.8 months, respectively. There was no significant difference between both groups except for the resection margins. The patients in the adjuvant CCRT group tended to have more positive resection margins (*p* = 0.007). The baseline characteristics, including age, gender, performance status, comorbidity, tumor location, histologic features (e.g. histology, grade, lymphovascular invasion, and perineural invasion), pathologic stage, LN status, and the preoperative CA19–9 levels, of the two groups were similar (Table 1).

The patterns of recurrence were evaluated for all patients. A total of 73 recurrences (79.3%) were observed. Locoregional recurrence occurred in 22 patients (23.9%), and distant recurrence occurred in 36 patients (39.1%). Local and distant recurrence occurred simultaneously in 15 patients (16.3%). There was no difference in the recurrence patterns of both the adjuvant CCRT and chemotherapy groups (*p* = 0.123) (Table 1).

The median follow-up duration was 20.7 months. Death had occurred in 63 of 92 patients (68.5%) during the follow-up period. The median and mean RFS were 12.8 and 25.9 months, whilst the median and mean OS were 26.1 and 39.2 months.

### Adjuvant concurrent chemoradiotherapy versus chemotherapy

**Fig. 1:**
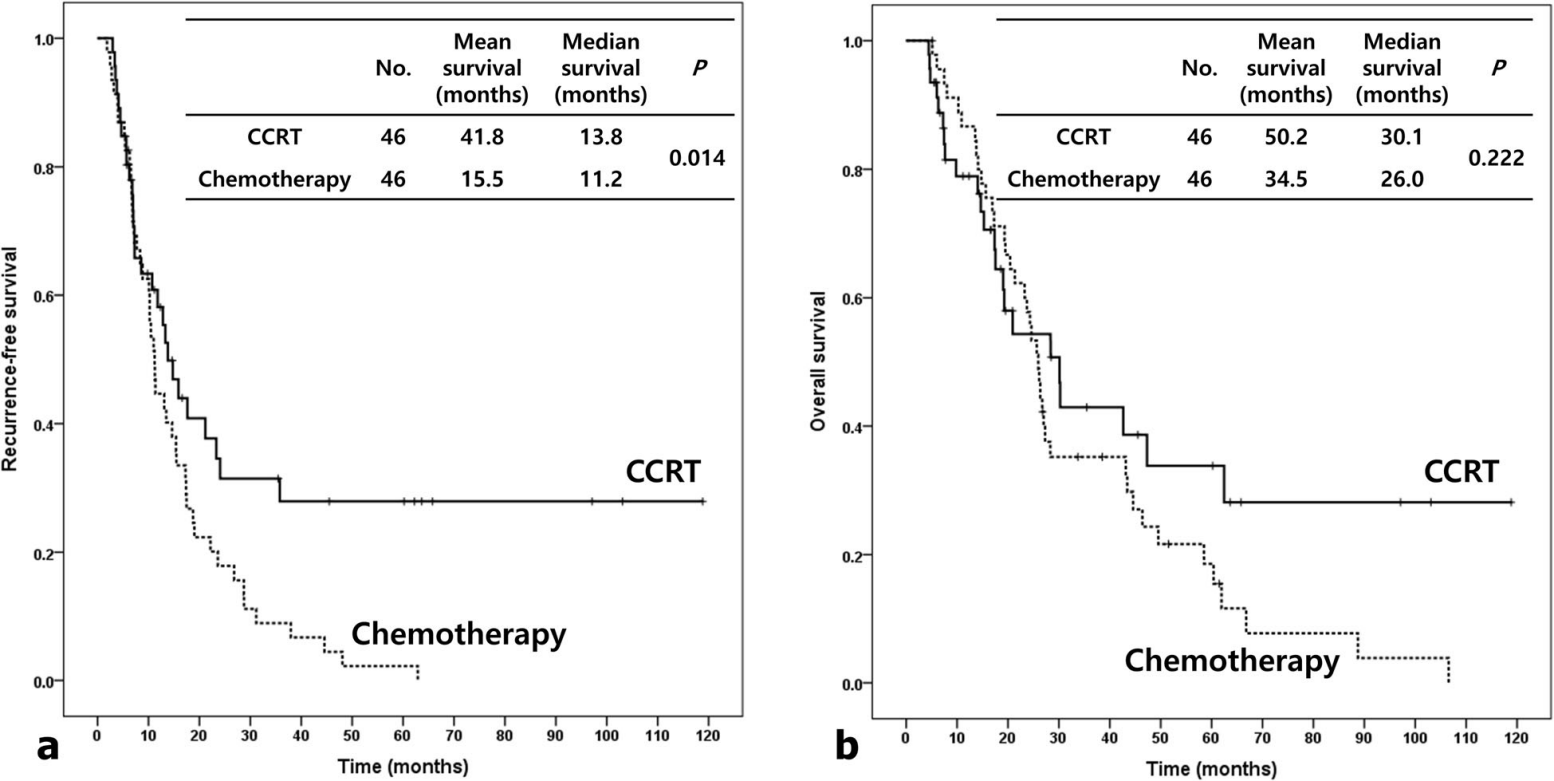
Recurrence-free survival and overall survival by adjuvant concurrent chemoradiotherapy (CCRT) and chemotherapy. Patients treated with adjuvant CCRT had better recurrence-free survival (**a**) and overall survival (**b**) as compared to those treated with adjuvant chemotherapy

**Fig. 2:**
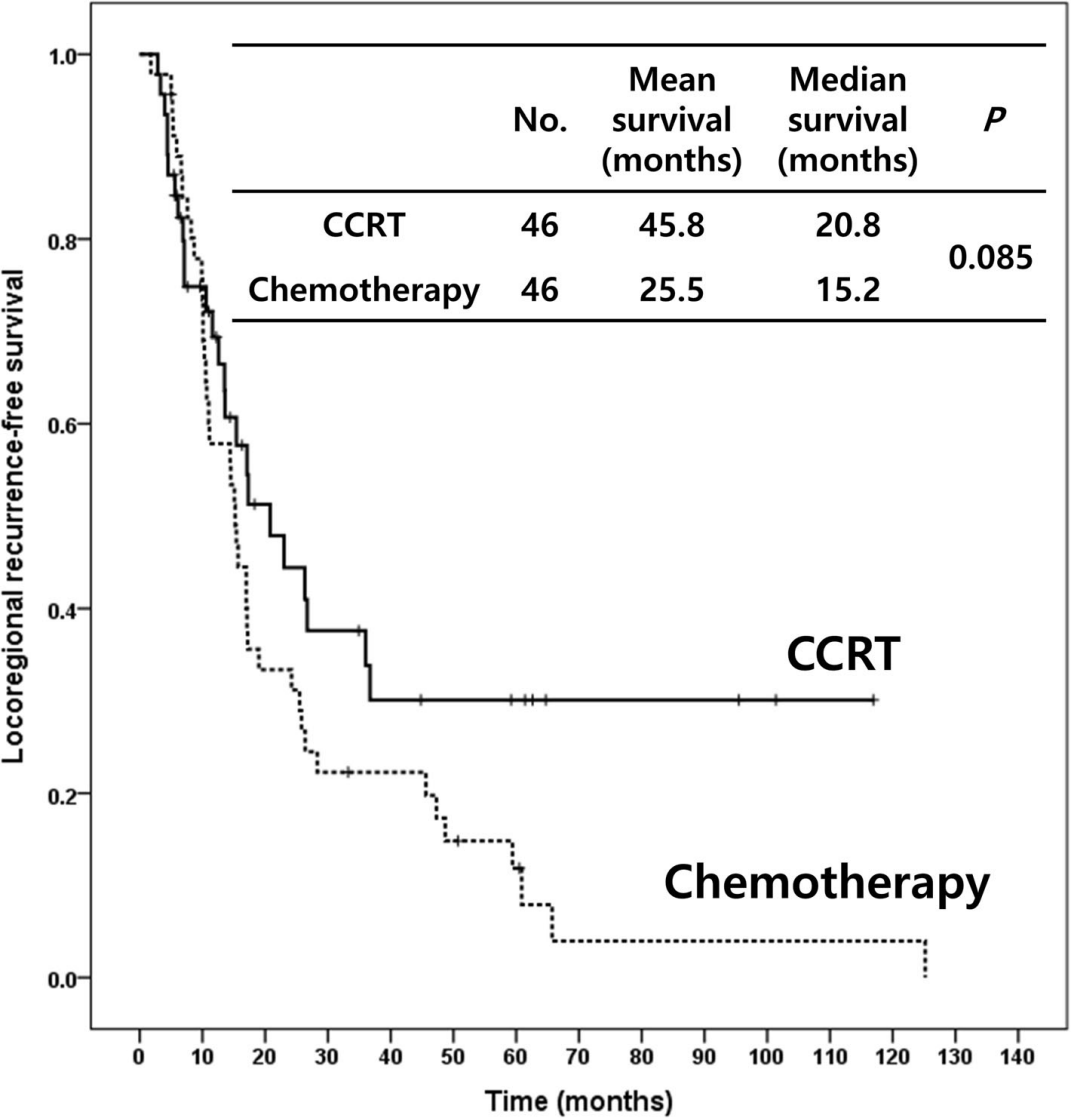
Locoregional recurrence-free survival by adjuvant concurrent chemoradiotherapy and chemotherapy

The median RFS for the adjuvant CCRT and chemotherapy groups were 13.8 and 11.2 months (*p* = 0.014), respectively (Fig. 1a). The median OS were 30.1 and 26.0 months (*p* = 0.222), respectively (Fig. 1b). Adjuvant CCRT had significantly better RFS and numerically higher OS than did chemotherapy. The median locoregional RFS for the adjuvant CCRT and chemotherapy groups were 20.8 and 15.2 months (*p* = 0.085), respectively (Fig. 2).

**Fig. 3:**
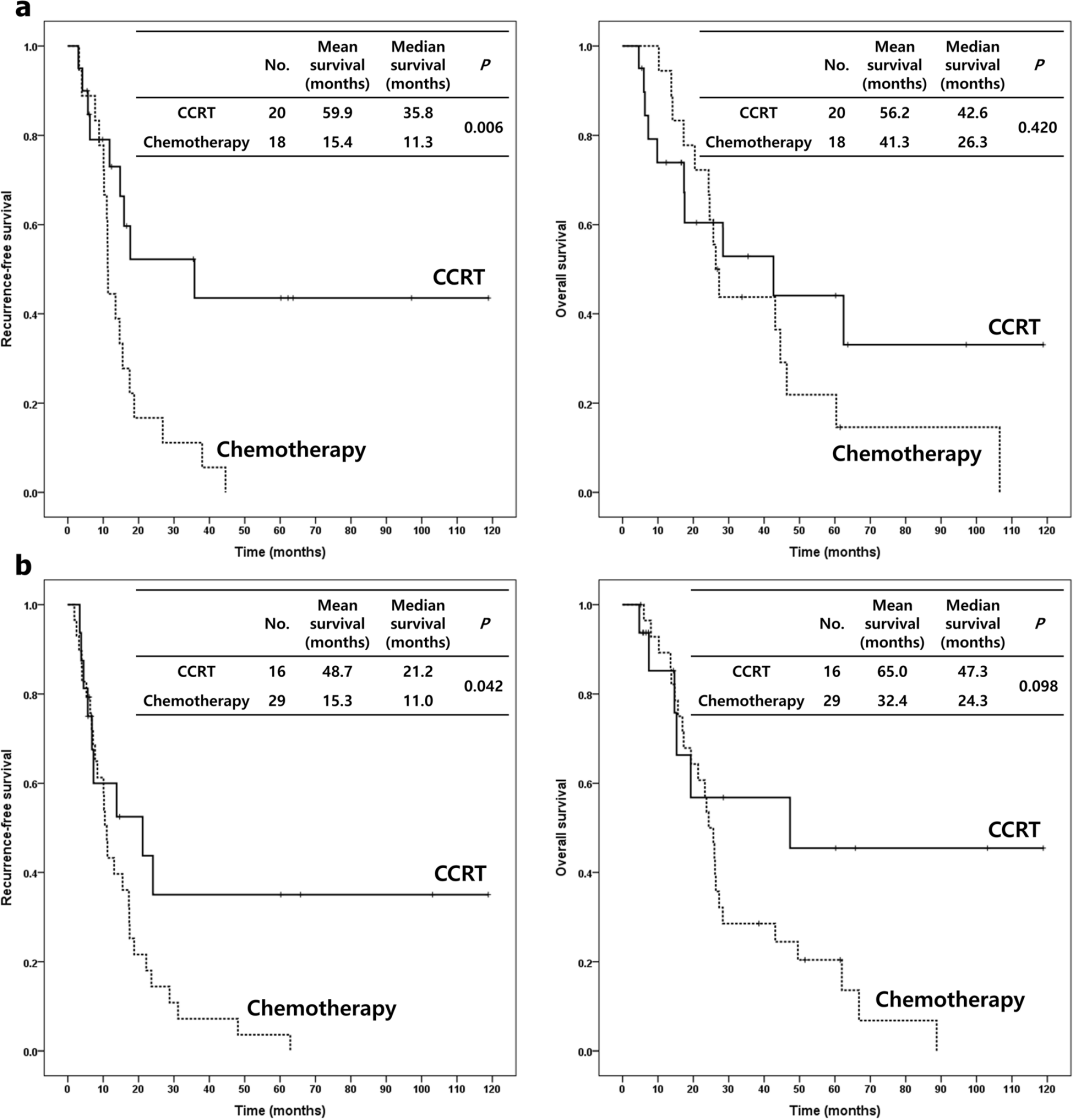
Recurrence-free survival and overall survival of negative lymph node involvement (**a**) and negative resection margin (**b**) groups by adjuvant concurrent chemoradiotherapy (CCRT) and chemotherapy. The patients treated with adjuvant CCRT showed better clinical outcomes than those treated with adjuvant chemotherapy, especially in negative lymph node involvement or negative resection margin groups

We performed a subgroup analysis comparing the RFS and OS of patients receiving adjuvant CCRT versus chemotherapy. In the subgroups with negative LN involvement (RFS *p* = 0.006, OS *p* = 0.420) (Fig. 3a) or negative resection margins (RFS *p* = 0.042, OS *p* = 0.098) (Fig. 3b), adjuvant CCRT led to significantly longer RFS and numerically higher OS than did chemotherapy. But adjuvant CCRT did not have more clinical benefits than did chemotherapy in the patients with positive LN involvement or positive resection margins. Also, adjuvant CCRT showed longer RFS than did chemotherapy in patients with well/moderate differentiated tumors (RFS *p* = 0.017, OS *p* = 0.580), pT1/2 (RFS *p* = 0.048, OS *p* = 0.248), or pStage I/II (RFS *p* = 0.018, OS *p* = 0.188), whereas there was less difference with regard to RFS and OS in patients with poorly differentiated tumors (RFS *p* = 0.448, OS *p* = 0.367), pT3/4 (RFS *p* = 0.180, OS *p* = 0.933), or pStage III/IV (RFS *p* = 0.391, OS *p* = 0.870). All other factors were not statistically different.

### Prognostic factors

For univariate analysis, the significant factors for RFS included perineural invasion (*p* = 0.033, HR 2.262) and pattern of adjuvant treatment (*p* = 0.016, HR 1.802). Histologic grade (*p* = 0.094, HR 1.606), lymphovascular invasion (*p* = 0.121, HR 1.528), and LN involvement (*p* = 0.063, HR 1.573) had no statistical significance, but poorly differentiated tumor or positive results tended to have poor prognoses (Table 2). For multivariate analysis, the pattern of adjuvant treatment, histologic grade (well/moderate/poor), lymphovascular invasion, perineural invasion, resection margin, LN involvement, pT stage (1/2 vs. 3/4), and pathologic stage (I/II vs. III/IV) were included in the model. Of these variables, the pattern of adjuvant treatment (CT vs. CCRT, *p* = 0.004, HR 2.351), histologic grade (poor vs. well, *p* = 0.023, HR 4.793), and LN involvement (*p* = 0.028, HR 1.912) remained significant factors for RFS. The resection margin (*p* = 0.076, HR 1.679) had borderline significance (Table 3).

**Table 2:** Univariate analysis for recurrence-free survival and overall survival

	OS			RFS		
	*P*	HR	95% CI	*P*	HR	95% CI
Age	0.099	1.524	0.924–2.512	0.622	1.123	0.708–1.781
Comorbidities						
CCI < 3	0.018	1.842	1.111–3.054	0.131	1.429	0.899–2.273
CCI ≥3						
Grade						
Well/Moderate	0.256	1.408	0.780–2.540	0.094	1.606	0.922–2.796
Poor						
Lymphovascular invasion	0.844	1.058	0.603–1.856	0.121	1.528	0.894–2.613
Perineural invasion	0.152	1.799	0.806–4.016	0.033	2.262	1.070–4.781
pT stage						
1–2	0.786	1.072	0.649–1.769	0.963	1.011	0.636–1.607
3–4						
Lymph node involvement	0.178	1.423	0.852–2.375	0.063	1.573	0.976–2.535
Pathologic stage						
I-II	0.588	1.150	0.694–1.907	0.495	1.175	0.739–1.868
III-IV						
Resection margin						
Negative	0.728	1.092	0.665–1.793	0.715	0.918	0.579–1.454
Positive						
Treatment						
Adjuvant CCRT	0.224	1.378	0.822–2.309	0.016	1.802	1.118–2.904
Adjuvant chemotherapy						

*OS* Overall survival, *RFS* Recurrence-free survival, *HR* Hazard ratio, *CCI* Charlson comorbidity index, *CCRT* Concurrent chemoradiation therapy

**Table 3:** Multivariate analysis for recurrence-free survival

	*P*	Hazard ratio	95% CI
Adjuvant chemotherapy (versus CCRT)	0.004	2.351	1.320–4.187
Grade (versus Well)			
Moderate	0.081	2.943	0.877–9.875
Poor	0.023	4.793	1.243–18.487
Resection margin (+)	0.076	1.679	0.948–2.974
LN involvement (+)	0.028	1.912	1.074–3.404

*CCRT* Concurrent chemoradiation therapy, *LV* Lymphovascular, *LN* Lymph node

## Discussion

BTC have a poor prognosis with high recurrence rates even after curative resection [5, 6, 8]. To reduce the recurrence rates, a strategy aimed at optimizing local and systemic controls may improve long-term survival outcomes [13]. Although the data and guidelines have supported an adjuvant approach, the choice of adjuvant therapy, which gives the best survival benefit, is controversial [11, 12, 14]. Therefore, this study was conducted to evaluate the effects of adjuvant CCRT and compared them to those of chemotherapy after radical resection in BTC.

Several retrospective and small prospective studies have shown adjuvant CCRT and chemotherapy to have benefits as compared to surgery alone or adjuvant radiotherapy. A meta-analysis from Horgan et al. [13] in 2012 included 6712 patients with resected cholangiocarcinoma, for whom adjuvant chemotherapy (*P* < 0.001, OR 0.39) and adjuvant chemoradiotherapy (*P = 0.049, OR 0.61)* had significantly improved OS more than had adjuvant radiotherapy alone (*P* = 0.90, OR 0.98). Also, a retrospective study in 2015 of 296 patients compared the effects of adjuvant chemotherapy and CCRT on BTC [15]. Both adjuvant therapies were associated with an OS benefit (*P* = 0.02, HR 0.41), especially for patients with R1 resection (*P* < 0.05, HR 0.23) and positive LN disease (*P* < 0.05, HR 0.46). In 2016, Kim et al. [16] conducted a study on the status of 158 patients after R0 resection of extrahepatic cholangiocarcinoma and compared the effects of adjuvant therapy, which demonstrated significant improvement in OS after chemotherapy (*P* = 0.001, HR 0.21) and chemoradiotherapy (*P* = 0.024, HR 0.25). A recent and small prospective multi-institutional phase II trial included patients with resected gallbladder cancer or extrahepatic cholangiocarcinoma, pT2-T4 or LN involvement or R1 resection status. This trial proposed gemcitabine/capecitabine chemotherapy followed by capecitabine CCRT as a promising adjuvant regimen [17].

Some published data have compared the benefits of adjuvant chemoradiotherapy and chemotherapy. A subgroup analysis by Nassour et al. [7] included data comparing the OS of patients who had received adjuvant chemoradiotherapy to those who had received chemotherapy for resected perihilar cholangiocarcinoma. There was a marginal OS benefit associated with the use of adjuvant chemoradiotherapy (mean OS 25 vs. 31 months, *P* = 0.04, HR 0.80). In this study, we reviewed 92 patients treated with adjuvant therapy in resected BTC and conducted a direct comparison with adjuvant CCRT and chemotherapy. As a result, adjuvant CCRT was associated with more improved RFS and OS than was chemotherapy in patients with resected BTC.

The high-risk factors, including LN involvement and positive resection margin, are commonly used to select patients with BTC for adjuvant therapy. Krasnick et al. [18] reported that adjuvant therapy is significantly associated with improved survival in perihilar cholangiocarcinoma patients with LN involvement. A meta-analysis of previous studies concluded that any adjuvant therapy had a significant benefit in patients with R1 resection or positive LN disease [13]. The NCCN guidelines recommend adjuvant therapy for node-positive disease and positive resection margins [14]. In the present study, we performed a subgroup analysis to determine the patient group benefiting the most from adjuvant CCRT. The gap of survival benefit between adjuvant CCRT and chemotherapy was significant among patients with negative resection margins or negative LN involvement. Because our results do not correspond with previous studies, the implications should be considered. With the assumption that the recurrence rates of locoregional diseases were high and their control improved survival, our institution has provided adjuvant CCRT or chemotherapy for locoregionally advanced diseases (LN involvement) or microscopic residual disease (R1 resection) after surgery [19–21]. Therefore, most of the patients included in this study were high-risk. A few patients with negative LN involvement and negative resection margins were included. On the other hand, adjuvant CCRT did not exhibit more clinical benefits than did chemotherapy for patients with positive LN involvement or positive resection margins. The patients included both positive groups. This result may imply that very high-risk patients with both positive resection margins and positive LN involvement had survival rates that were too poor to exhibit a difference between CCRT and chemotherapy, similar to patients with poorly differentiated tumors and higher pathologic stages. This outcome suggests a need for the graded classification of risk factors. Such a classification could recommend adequate intermediate-risk groups for adjuvant CCRT.

The prognostic factors for resected BTC have been identified in many studies. Lim et al. [10] discovered significant prognostic factors, such as the pattern of adjuvant treatment, elevated CA 19–9 levels, and histologic grade. Also, Leng et al. [22] revealed that an advanced tumor stage, positive LN, and poorly differentiated tumors were significantly associated with poor survival in resected perihilar cholangiocarcinoma. Most of the studies included adjuvant therapy, histologic grade, LN status, and tumor stage as prognostic factors. In the present study, the pattern of adjuvant treatment, histologic grade, LN involvement, and resection margin were significant prognostic factors for RFS on multivariate analysis. Our data are in line with those of previous studies.

There are several limitations to this study. First, because of its retrospective nature and the non-random distribution in adjuvant therapy, the outcomes could be influenced by selection bias. Second, our study included diverse chemotherapy regimens, such as monotherapy or combination therapy, intravenous or oral agents, and the duration of chemotherapy. The effects of these differences on the results are unknown. Third, we could not account for the differences among the tumor locations of BTC; the gallbladder cancer and the intrahepatic, perihilar, and distal extrahepatic cholangiocarcinoma. Finally, due to considerable patient and treatment heterogeneity, a relatively long study period may also influence clinical outcomes. However, this study provides important information about the selection of adjuvant therapy in resected BTC.

## Conclusions

Our study has demonstrated the superiority of adjuvant CCRT over chemotherapy for improving RFS in curatively resected BTC. We found that adjuvant CCRT was more effective treatment than chemotherapy in subgroup patients, especially those with negative LN involvement, negative resection margins, lower stage, or better tumor grade. Also, our study showed that the pattern of adjuvant treatment, histologic grade, LN involvement, and resection margin were significant prognostic factors. Our results could help researchers establish the application of adjuvant CCRT. Further prospective studies are needed to evaluate the efficacy of adjuvant CCRT for BTC.

## Data Availability

The datasets generated and/or analyzed during the current study are not publicly available but are available from the corresponding author on reasonable request.

## Abbreviations

5-FU: 5-fluorouracil
AJCC: American Joint Committee on Cancer
BTC: Biliary tract cancers
CA19–9: Carbohydrate antigen 19–9
CCI: Charlson comorbidity index
CCRT: Concurrent chemoradiotherapy
CI: Confidence intervals
HR: Hazard ratios
KPS: Karnofsky performance status
LN: Lymph node
NCCN: National Comprehensive Cancer Network
OS: Overall survival
RFS: Recurrence-free survival
