# Correction to: Comparison of the effects of adjuvant concurrent chemoradiotherapy and chemotherapy for resected biliary tract cancer

**DOI:** 10.1186/s12876-020-01347-3

**Published:** 2020-07-02

**Authors:** Hyera Kim, Mi Hwa Heo, Jin Young Kim

**Affiliations:** Division of Hematology and Oncology, Department of Internal Medicine, Keimyung University, Dongsan Medical Center, 56 Dalseong-ro, Jung-gu, Daegu, 41931 South Korea

**Correction to: BMC Gastroenterol (2020) 20:20**

**https://doi.org/10.1186/s12876-020-1171-1**

Following publication of the original article [1], we have been notified that there is a mistake in the corresponding author mentioned.

Currently the corresponding author mentioned is: Hyera Kim (kheyra@naver.com). However, it should be Jin Young Kim (takgu@dsmc.or.kr)
